# Atrial Septal Defect Presenting as Fleischner's Sign in an Elderly Patient: A Case Report

**DOI:** 10.7759/cureus.83407

**Published:** 2025-05-03

**Authors:** Yuki Ohnishi, Lusine Zakharian, Yasuhiro Suyama, Hiroyuki Otsuka

**Affiliations:** 1 Internal Medicine, Sosa Municipal Hospital, Chiba, JPN; 2 Internal Medicine, 374th Medical Group Yokota Air Base Hospital, Tokyo, JPN; 3 Rheumatology, Nippon Telegraph and Telephone (NTT) Medical Center Tokyo, Tokyo, JPN

**Keywords:** atrial septal defect, chest x-ray, congenital heart disease, health check-up, incidental radiological finding

## Abstract

In the elderly, the most common congenital heart disease is secundum atrial septal defect (ASD). We present a case of an elderly patient with an incidental diagnosis of asymptomatic ASD, detected on a posteroanterior chest X-ray by Fleischner’s sign. Fleischner’s sign is defined as the radiographic finding of a prominent central pulmonary artery, suggesting pulmonary hypertension. This is rarely seen in ASD that remains undiagnosed until elderhood. Although congenital heart disease is relatively rare in the elderly, physicians should maintain a high index of suspicion when interpreting chest X-rays that exhibit the Fleischner’s sign in the hila of the lungs.

## Introduction

Atrial septal defect (ASD) is the most common congenital cardiac anomaly presenting in adulthood; however, it often manifests with atypical clinical symptoms and misleading auscultatory findings [1]. We report a unique case of an asymptomatic elderly patient whose health check-up chest X-ray incidentally revealed the Fleischner’s sign, ultimately leading to a diagnosis of longstanding, unrecognized ASD. Fleischner’s sign describes a prominent central pulmonary artery, often resulting from pulmonary hypertension (PAH) or vessel distension [2]. This finding was first reported in a case of pulmonary embolism and is suggestive of PAH; it has also been observed in patients with ASD. Although the Fleischner’s sign is rare in patients with ASD unless the defect remains undiagnosed for several decades, recognition of this characteristic radiographic sign on chest X-ray, a widely available and commonly used imaging modality, may facilitate improved diagnosis of ASD.

## Case presentation

Our patient was an 80-year-old man with a history of hypertension and benign prostatic hyperplasia, both well-controlled on medication. He was referred to our hospital for a pre-admission health check-up before entering a nursing home. He was asymptomatic and denied a history of smoking or a family history of cardiac disease.

**Figure 1:**
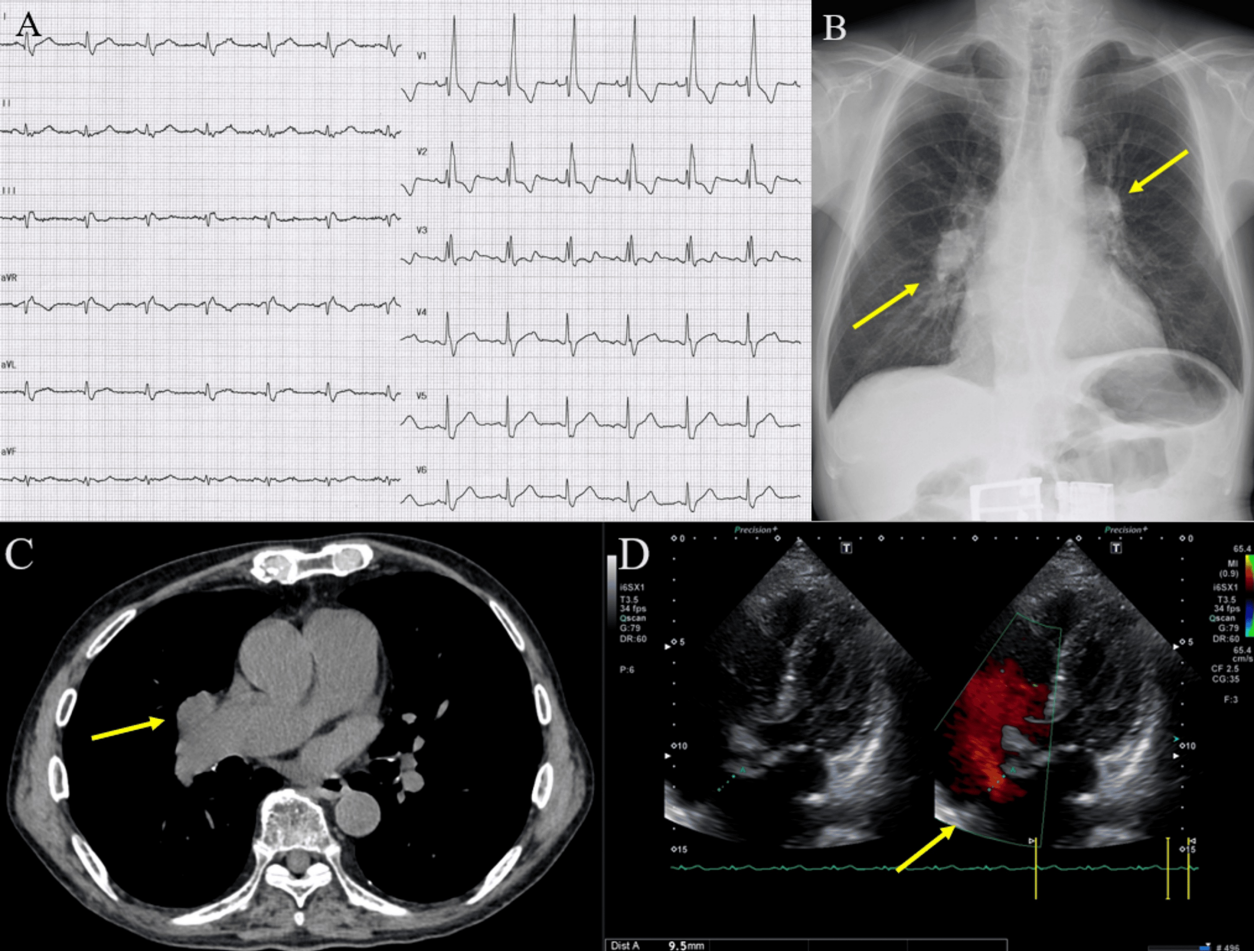
Actual electrocardiogram, chest radiography, CT scan of the chest, and echocardiogram images 1A: Electrocardiogram showing complete right bundle branch block; 1B: Chest X-ray showing opacity in the hila of the lungs, consistent with “Fleischner’s sign” (arrows); 1C: Chest computerized tomography demonstrating dilated pulmonary hilar vessels; 1D: Transthoracic echocardiography with color flow Doppler showing the atrial septal defect with a 10 mm distinct jet seen crossing the septum from left to right.

On examination, his blood pressure was 126/74 mmHg, his heart rate was 78 beats per minute, and oxygen saturation (SpO₂) was 98% on room air. Physical examination revealed no signs of congestive heart failure and no clear fixed splitting of the second heart sound on auscultation. The electrocardiogram showed complete right bundle branch block (Figure 1A). Chest X-ray was revealing for Fleischner’s sign in the hila of the lungs (Figure 1B). A subsequent CT of the chest also displayed similar findings, which excluded the possibility of a lung mass (Figure 1C). Transthoracic echocardiography (Figure 1D) revealed a 9.5 mm diameter secundum ASD with an estimated pulmonary/systemic flow ratio of 1.04. The echocardiogram showed a transtricuspid pressure gradient (TRPG) of 23 mmHg but no signs of right ventricular overload or severe valvular heart disease at this point. He declined further invasive evaluation or treatment due to advanced age and mild symptoms. One year later, he presented with dyspnea and worsening bilateral lower extremity edema, necessitating diuretic therapy for heart failure management.

## Discussion

Congenital heart diseases diagnosed via autopsy in Japanese individuals aged 60 and over have been reported at 1.1%, with secundum ASD being the most common [3]. A previous study has shown that one-quarter of patients with ASD die before the age of 27, half before the age of 36, three-quarters before 50, and 90% before 60 years, with the median age of death being 37 years [4].

In Japan, health check-ups are conducted multiple times during the school-age years. As a result, congenital heart diseases that would be candidates for treatment are often presumed to be diagnosed in childhood. There seems to be less attention to congenital heart diseases during adult health check-ups [5]. In contrast, the rate of newly diagnosed congenital heart diseases in health check-ups targeting adults is 0.046%, nearly equivalent to the rate of new discoveries in school-age heart examinations, which is 0.04% [5]. In cases of atrial septal defects in adults, the reported occurrence frequency of fixed split-second heart sounds and mid-diastolic murmurs is low [6]. In the elderly, congenital heart disease is relatively rare, but it should be considered in the differential diagnosis when encountering unexplained heart failure or arrhythmia, with chest X-ray being an essential part of the diagnostic evaluation.

Fleischner’s sign is named after American radiologist Felix Fleischner, who first described it in 1961 [2]. This radiographic sign describes a prominent central pulmonary artery, typically seen in the setting of massive pulmonary embolism or PAH, often resulting from vessel distension due to a large pulmonary embolus affecting at least 50% of the major pulmonary artery branches [2]. Fleischner’s sign, found on chest X-ray from symptomatic ASD, has been anecdotally reported [7,8]. Our case involved an asymptomatic elderly male patient with ASD who exhibited the Fleischner's sign on chest X-ray and subsequently developed symptoms of heart failure. It is reported that atypical findings were more common in patients over the age of 50 due to various factors, including the development of noncompliance and increased pressure in the right atrium. In small defects, there may be increased left atrial pressure due to left ventricular failure and mitral valve disease [2]. For this reason, our case might exhibit the Fleischner's sign on the chest X-ray with a smaller defect compared to the previous case. Also, it is hypothesized that closure of ASD may be beneficial even in patients aged 60 years or older [9]; therefore, timely diagnosis of ASD in adults and elderly patients is important to prevent complications such as heart failure and to improve cardiac outcomes.

## Conclusions

Congenital heart diseases that remain undiagnosed into adulthood and elderhood often present without typical findings, making diagnosis challenging, especially in elderly patients. Although characteristic findings in elderly patients with undiagnosed ASD have not been well described, Fleischner’s sign offered a valuable diagnostic clue in our case. Even in cases without the heart murmurs well-known for ASD, clinicians should be aware of the value of Fleischner’s sign in discovering ASD. Utilizing this radiographic sign may help the detection of overlooked ASD, especially when advanced diagnostic tools are unavailable, thereby potentially improving patient outcomes.
